# Alkyl Protocatechuate-Loaded Nanostructured Lipid Systems as a Treatment Strategy for *Paracoccidioides brasiliensis* and *Paracoccidioides lutzii In Vitro*

**DOI:** 10.3389/fmicb.2017.01048

**Published:** 2017-06-12

**Authors:** Kaila P. Medina-Alarcón, Junya L. Singulani, Aline R. Voltan, Janaina C. O. Sardi, Maicon S. Petrônio, Mariana B. Santos, Carlos R. Polaquini, Luis O. Regasini, Vanderlan S. Bolzani, Dulce H. S. da Silva, Marlus Chorilli, Maria J. S. Mendes-Giannini, Ana M. Fusco-Almeida

**Affiliations:** ^1^Mycology Laboratory and Nucleus of Proteomics, Department of Clinical Analysis, School of Pharmaceutical Sciences, São Paulo State UniversityAraraquara, Brazil; ^2^Department of Chemistry and Environmental Sciences, Institute of Biosciences, Humanities and Exact Sciences, São Paulo State University, São José do Rio PretoAraraquara, Brazil; ^3^Department of Chemistry, Institute of Chemistry, São Paulo State UniversityAraraquara, Brazil; ^4^School of Pharmaceutical Sciences, Department of Drugs and Medicines, São Paulo State UniversityAraraquara, Brazil

**Keywords:** dodecyl, nanostructured lipid system, *Paracoccidioides brasiliensis*, *Paracoccidioides lutzii*, antifungal

## Abstract

Dodecyl protocatechuate (dodecyl) is a derivative of protocatechuic acid (3,4-dihydroxybenzoic acid) that possesses anti-oxidant and antifungal properties. Nanostructured lipid systems (NLS) can potentiate the action of many antifungal agents, reducing the required dose and side effects by improving their activity. This work aimed to evaluate dodecyl protocatechuate loaded into a NLS (NLS+dodecyl) as a strategy for the treatment of *Paracoccidioides brasiliensis* and *P. lutzii in vitro.* Antifungal activity against *P. brasiliensis* and *P. lutzii* was evaluated using the microdilution technique. NLS+dodecyl showed high antifungal activity with a minimum inhibitory concentration ranging from 0.06 to 0.03 μg/mL; 4- to 16-fold higher than that of free dodecyl. NLS+dodecyl was able to inhibit fungal adhesion of the extracellular artificial matrix proteins (laminin and fibronectin), resulting in 82.4 and 81% inhibition, respectively, an increase of 8–17% compared with free dodecyl. These findings corroborate previous results demonstrating 65 and 74% inhibition of fungal adhesion in pulmonary fibroblast cells by dodecyl and NLS+dodecyl, respectively, representing a 9% increase in inhibition for NLS+dodecyl. Subsequently, cytotoxicity was evaluated using the 0.4% sulforhodamine B assay. NLS+dodecyl did not exhibit cytotoxicity in MRC5 (human pneumocyte) and HepG2 (human hepatic carcinoma) cells, thus increasing the selectivity index for NLS+dodecyl. In addition, cytotoxicity was evaluated *in vivo* using the *Caenorhabditis elegans* model; neither dodecyl nor NLS+dodecyl exhibited any toxic effects. Taken together, these results suggest that NLS can be used as a strategy to improve the activity of dodecyl against *P. brasiliensis* and *P. lutzii* because it improves antifungal activity, increases the inhibition of fungal adhesion in lung cells and the extracellular matrix *in vitro*, and does not exhibit any toxicity both *in vitro* and *in vivo*.

## Introduction

Paracoccidioidomycosis, which is one of the most important systemic mycoses in Latin America, is a disease caused by the dimorphic fungi *Paracoccidioides brasiliensis* and *P. lutzii*. PCM is found from southern Mexico to northern Argentina and especially in Brazil, where the greatest number of cases have been reported (San-Blas et al., 2002; Colombo et al., 2011; Hernández et al., 2011; Alvarenga et al., 2016). Human infection by *Paracoccidioides spp.* occurs primarily through the respiratory system via inhalation of spores in the mycelial form, which transform into yeast following an increase in temperature. The disease can develop immediately following infection or even years later, depending on fungal virulence, its ability to interact and invade the surface of the cell, and the host immune response (Mendes-Giannini et al., 2000; Marcos et al., 2012; de Souza et al., 2014; de Oliveira et al., 2015). Virulence factors can be divided into two categories: those that promote colonization and invasion and those that harm the host such as toxins and proteinases. Adherence refers to pathogen recognition of ligands on the surface of the host cell or constituents of the basement membrane such as fibrinogen, laminin, collagen, and fibronectin. Several types of collagen are present in the interstitium, particularly as part of fiber lattices, which constitute target molecules for fungal, parasite, and bacterial infection (González et al., 2005; Marcos et al., 2012; de Oliveira et al., 2015).

Amphotericin B (AMB) is a drug of first choice for severe PCM, regardless of the immune status of the patient. AMB causes many side effects, such as nephrotoxicity, in patients, thus making conventional therapy difficult (Shikanai-Yasuda et al., 2006; Bocca et al., 2013). Other treatments, such as the use of corticosteroids, have been reported as adjuvant therapies in the treatment of infectious diseases, including PCM; these aim to reduce the inflammatory response of the host, which can result in tissue damage and organic dysfunction (Benard et al., 2012). Ketoconazole is classified as moderately effective for mycosis treatment; it was replaced by ITC because of its low absorption and adverse effects. Although ITC is more active, it produces many drug interactions and consequently causes several side effects (Naranjo et al., 2010; Shikanai-Yasuda, 2015). Antifungal resistance and at-risk patient populations are increasing. Furthermore, the number of commercially available antifungal drugs, many of which still present numerous side effects, is restricted. Together, these issues emphasize the need to develop new antifungal substances from natural sources (Nishioka, 1996; Lortholary et al., 1999; Shikanai-Yasuda, 2015).

**FIGURE 1:**
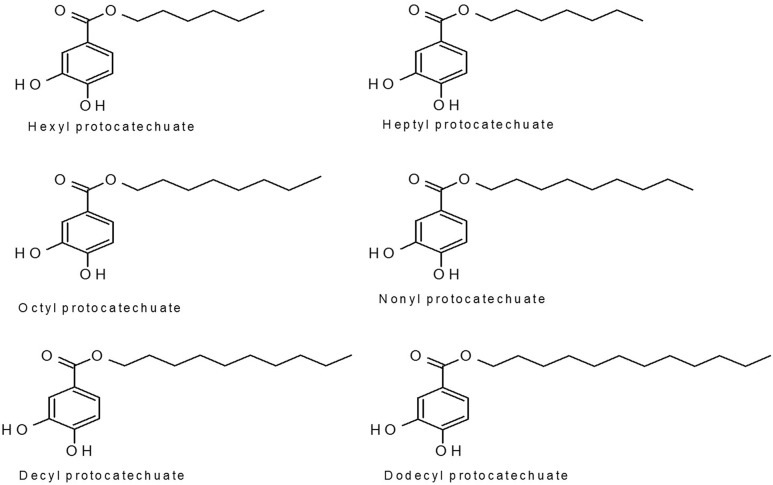
Protocatechuate structures.

Protocatechuic acid, also known as 3,4-dihydroxybenzoic acid, is found in fruits, nuts, and vegetables including olives, rice, tea, white wine, and herbal medicines. Their alkyl derivatives are shown in **Figure 1**. Phenolic compounds have been widely studied for their pharmacological activities and for their ability to inhibit lipid oxidation and the proliferation of fungi (Lin et al., 2009; Soares et al., 2014; Khan et al., 2015). The chemical complexity of compounds is important for their incorporation into a vehicle that will enable release of the active ingredient and the biological response (Bonifácio et al., 2015).

Technological strategies, including NLSs, such as microemulsion, offer efficient compartmentalization and oral bioavailability and improve the solubility of several active groups. Lipid-based drug delivery systems are used in the development of products because of their versatility with pharmaceutical excipients and their compatibility with liquid, semi-solid, and solid pharmaceutical forms (Cerpnjak et al., 2013).

Previous studies using AMB have shown that the presence of lipids in nanoemulsions and microemulsions enabled their successful delivery in respiratory therapy (Nasr et al., 2012; Hussain et al., 2014). Other lipid formulations of AMB are used to treat severely disseminated infections such as PCM, histoplasmosis, mucormycosis, and cryptococcosis (Roberts et al., 2015).

In addition, a recent study of compound and nanoparticle toxicity was performed in the *in vivo* model *Caenorhabditis elegans* (Okoli et al., 2009; Contreras et al., 2014; Rogers et al., 2015; Muhammed et al., 2016). This free-living nematode presents various advantages such as a short life cycle (3–4 days at 20°C), small size (1 mm), transparency, easy cultivation, and a well-characterized genome (Kaletta and Hengartner, 2006; Peterson et al., 2008).

The aim of this study was to investigate the effects of dodecyl protocatechuate-loaded in NLS (NLS+dodecyl) as a strategy for improving the antifungal activity of free dodecyl protocatechuate against *Paracoccidioides* spp. *in vitro*. Moreover, we evaluated the toxicity of free and NLS-incorporated dodecyl protocatechuate in MRC5 and HepG2 cells and in the *C. elegans* model.

## Materials and Methods

### Compounds

Alkyl derivatives of protocatechuic acid were prepared using esterification reactions as described by de Faria et al. (2012) with minor modifications. Three milliliters of N,N′-dicyclohexylcarbodiimide (DCC, 1.0 mmol) in dry p-dioxane was added to 0.2 mmol of cooled (5°C) protocatechuic acid and 20 mmol of n-alkyl in 6.0 mL of dry p-dioxane. After 48 h, the solvent was removed under reduced pressure. The residue was partitioned with EtOAc (3× EtOAc) and filtered. The filtrate was washed successively with a saturated aqueous citric acid solution (3×) and with saturated aqueous NaHCO_3_ (3×). The organic phase was dried over anhydrous MgSO_4_ and evaporated under reduced pressure. The crude products were purified over a silica gel column and eluted isocratically with CHCl_3_/MeOH (98:2) to yield esters. Their structures were confirmed by ^1^H and ^13^C NMR spectral analyses. The substances were dissolved in 1 to 3% DMSO (Soares et al., 2014).

### Microorganisms

*Paracoccidioides brasiliensis* S1 isolate 18 (Pb18-chronic PCM/São Paulo, Brazil), *P. brasiliensis* S2 (02-chronic PCM/Venezuela), and *P. lutzii* Pb01-strain ATCC MYA-826 (acute PCM/Goiânia, Brazil) were obtained from the mycology collection of the Mycology Laboratory, School of Pharmaceutical Sciences and were maintained in Fava-Netto agar medium at 37°C.

### Minimum Inhibitory Concentration (MIC)

The MIC assay was performed according to the microdilution method described by Clinical and Laboratory Standards Institute (CLSI, 2008) document M27-A3 with modifications (de Paula e Silva et al., 2013). Alkyl derivatives of protocatechuic acid (hexyl, heptyl, octyl, nonyl, decyl, and dodecyl protocatechuates) were diluted according to CLSI M27-S3. The yeast suspension was adjusted to a final concentration of 1.5 × 10^3^ CFU/mL in RPMI-1640 medium (Sigma–Aldrich, St. Louis, MO, United States) in 96-well plates and serial dilutions (from 250 to 0.24 μg/mL) of the compounds were then added; AMB and ITC were used as controls. The plates were incubated on a shaker at 37°C/150 rpm for a specific duration individually determined for each microorganism. The MIC was determined using alamar blue (Sigma–Aldrich, St. Louis, MO, United States) at 530 and 570 nm; a change from the original blue color of the reagent to pink confirmed death or growth inhibition induced by the antifungal compounds. Following 72 or 96 h of incubation. The Minimum Fungicide Concentration (MFC) was determined by transferring an aliquot from the wells to Brain Heart Infusion (BHI) medium supplemented with 2% glucose (Difco, BD Biosciences) plates, which were then incubated at 37°C for 5–7 days. The MFC was defined as the concentration that inhibited growth (Soares et al., 2014).

### Cytotoxicity Assay

The cytotoxicity of the protocatechuates was assessed in the MRC5 cell line (MRC5) obtained from the American Type Culture Collection (ATCC; Manassas, VA, United States) and HepG2 cell line obtained from the Biotechnology Laboratory Biotech (UNICAMP, Brazil). The plates were first incubated with 1 × 10^5^ cells per well at 36.5°C under 5% CO_2_ for 24 h to form a cell monolayer (Husøy et al., 1993). Compounds were tested at different concentrations ranging from 0.12 to 62.5 μg/mL using serial dilutions in a total volume of 100 μL per well. After the 24 h treatment the cytotoxicity was assessed by the 0.4% sulforhodamine B (SRB) (Vichai and Kirtikara, 2006). The assay is sensitive and measures the cellular protein content of adherent or suspension cultures in 96-well plates. Cells were fixed with trichloroacetic acid (TCA) and then stained for 30 min with SRB dissolved in acetic acid. In mildly acidic conditions, SRB binds on basic amino acid residues on TCA-fixed cells. The unbound dye is then removed after washing and the dye bound to the protein is solubilized in basic medium (Tris-base). The absorbance was measured at 570 nm. The result of the percentage of viable cells was performed using the following formula: according to Rajpoot et al. (2012) with minor modification.

Viable cells % = (test mean - hite mean/mean negative control - mean white) × 100

### Preparation of the Nanostructured Lipid System (NLS)

The NLS was prepared according to previously published methodology with modifications (Formariz et al., 2008, 2010). The NLS is formed using cholesterol (10%) as the oily phase; phosphate buffer (pH 7.4; 80%) as the aqueous phase; and mixed non-ionic surfactant castor oil, polyoxyl-60/PEG-hydrogenated, PS and OS (8: 6: 3 ratio and a final concentration of 10% of the formulation). The mixture was sonicated (Sonics^®^ Vibra-Cell) at 220 W for 30 min in the discontinuous mode with a 30 sec interval in an ice bath. Following sonication, the NLS was centrifuged at 11,180 ×*g* for 15 min to eliminate the residue released by the titanium rod sonicator. The samples were characterized as a liquid that was optically translucent or opaque.

### Preparation of NLS+Dodecyl

Following NLS preparation, 2 mg of dodecyl protocatechuate (dodecyl) was incorporated into 2 mL of NLS, resulting in NLS+dodecyl at a final concentration of 1mg/mL, which was homogenized and sonicated for 20 min under the same conditions used in NLS preparation. Subsequently, the NLS+dodecyl was centrifuged at 11,180 ×*g* for 15 min to eliminate the residue released by the titanium rod sonicator. The formulations were then passed through a 0.22 μm sterile filter (Millipore). The mean diameter, PDI, and zeta potential of the NLS and NLS+dodecyl were characterized by using the Zetasizer Nano NS model (Malvern Instruments, Malvern, United Kingdom).

### Characterization of the Physicochemical Properties of the Nanoparticles: Determination of Diameter Mean, PDI, and Zeta Potential

Droplet diameter, PDI, and zeta potential, were determined with and without dodecyl. All samples were diluted: 10 μL of sample in 900 μL of deionized water. The samples were analyzed using dynamic light scattering with a wavelength of 633 nm at both 25°C and an angle of 90° at 25°C with a Zetasizer Nano ZS particle analyzer (Malvern Instruments, Malvern, United Kingdom) coupled to a multi-purpose titrator. Ten measurements were evaluated for diameter, PDI, and zeta potential; this assay was based on the Doppler Effect (Csaba et al., 2009).

### High Resolution Scanning Microscopy (FEG/SEM)

High resolution scanning microscopy was applied to determine the microstructure of the NLS and NLS+dodecyl. All sample was first lyophilized and suspended in water and then placed on a carbon-coated copper grid. The SEM images were obtained using a JEOL JSM-7500F microscope.

### Formation and Inhibition of *P. brasiliensis* 18 Adhesion to the Artificial Extracellular Matrix (ECM)

Initially, 24-well plates were coated with ECM of fibronectin and laminin, respectively (Sigma–Aldrich, St. Louis, MO, United States) at a concentration of 50 μg/mL for 18 h at 4°C followed by incubation for 1 h at 37°C. The plates were then washed three times with PBS and fungal suspension was added at a concentration of 1.5 × 10^3^ CFU/mL, which had been inoculated into the 24-well plate previously sensitized with ECM and incubated at 37°C for 1, 3, or 5 h. Subsequently, the supernatant was removed, the plate was washed with PBS, and then 300 μL of trypsin was added (Sigma). The supernatant was obtained and then centrifuged at 10,000 rpm for 5 min at (4°C). The new supernatant was discarded and 500 μL of FACS buffer was added for subsequent cell counting by flow cytometry (BD FACS Canto and BD FACS Diva software analysis). Following the adhesion period, treatment with the selected compound (dodecyl and NLS+dodecyl) was performed for 24 h at 37°C. RPMI-1640 with L-glutamine and 2% glucose was used to maintain adequate nutritional status. Subsequently, the supernatant was removed and the plate was washed with PBS. Then, 300 μL of trypsin was added and centrifuged. The supernatant was discarded and 500 μL of FACS buffer was added to each tube for subsequent cell counting by flow cytometry (BD FACS Canto and BD FACS Diva software analysis).

### Inhibition of *P. brasiliensis* 18 Adhesion to Cells

Cells (MRC5) were cultured at 36.5°C in 24-well plates and adjusted to 1 × 10^5^ cells per well. A *P. brasiliensis* suspension (1.5 × 10^3^ CFU/mL) was labeled with 10 μM of 5(6)-carboxyfluorescein diacetate *N*-succinimidyl ester (CFSE; Sigma–Aldrich) and then added to the cells. The plate was incubated for 3 h at 36.5°C under 5% CO_2_ to enable the adhesion process. Subsequent to treatment, dodecyl and NLS+dodecyl were applied and the plate was incubated for 24 h under the same conditions. The supernatant was removed and the plate was washed with PBS. Then, 300 μL of trypsin (Sigma–Aldrich,) was added and centrifuged. The supernatant was discarded and 500 μL of FACS buffer was added to each tube, for subsequent cell counting by flow cytometry (BD FACS Canto) and BD FACS Diva software analysis (Mendes-Giannini et al., 1994, 2004; Hanna et al., 2000).

### Toxicity Assay in *C. elegans* Model

*Caenorhabditis elegans* wild-type strain N2 was routinely cultured at 22°C on nematode growth medium (NGM) plates seeded with *E. coli* strain OP50. For the toxicity assay, eggs were harvested to obtain synchronous cultures. After 3 days, worms in the L4 stage were washed with M9 buffer. Then, approximately 20 worms were transferred to each well of a 96-wells plate containing 100 μL of 60% M9 buffer, 40% BHI, 10 μg/mL cholesterol (in ethanol), 200 μg/mL ampicillin, and 90 μg/mL kanamycin. Next, 100 μL of dodecyl protocatechuate, NLS, or NLS+dodecyl were added to the medium with final compound concentrations in each well ranging from 0.03 to 125 μg/mL. The plate was incubated at 22°C for 24 h. Worm survival was evaluated based on mobility and form (worms with a stick shape were considered dead and worms with a sinusoidal shape were considered alive). Images were acquired using an IN Cell Analyzer 2000 (GE Healthcare), microscope (10×).

### Statistical Analysis

All of the tests were performed as three independent experiments. Statistical analysis of the data was carried out by analysis of variance (ANOVA) with Bonferroni post-test at a 5% significance level. The results are expressed as the mean ± standard deviation (SD). The statistical software used was GraphPad Prism *5.0* (La Jolla, CA, United States).

## Results

### Minimum Inhibitory Concentration (MIC) of Protocatechuates

The antifungal activities of hexyl, heptyl, octyl, nonyl, decyl, and dodecyl protocatechuates were evaluated using *P. brasiliensis* and *P. lutzii*. The determined MICs ranged from 0.48 to 7.8 μg/mL for *P. lutzii* (Pb01) and *P. brasiliensis* (Pb18) and from 0.24 to 1.9 μg/mL for *P. brasiliensis* (Pb02). The MICs of AMB and ITC ranged from 0.02 to 0.12 μg/mL and from 0.008 to 0.12 μg/mL, respectively, for the three fungal strains. These results are detailed in **Table 1**. All protocatechuates showed potent antifungal activity; however, the strongest activity was observed for the dodecyl protocatechuate against the three phylogenetic species, with an MIC of 0.24–0.48 μg/mL.

**Table 1:** Anti-*Paracoccidioides* activity, cytotoxicity, and SI of different protocatechuate esters.

	Pb18 MIC	Pb01 MIC	Pb02 MIC	MRC5 IC_50_	SI	SI	SI
	(μg/mL)	(μg/mL)	(μg/mL)	(μg/mL)	(IC_50_/MIC Pb18)	(IC_50_/MIC Pb01)	(IC_50_/MIC Pb02)
hexyl	3.9	7.8	1.9	29.5	7.5	3.8	15.5
heptyl	7.8	3.9	0.24	17.6	2.3	4.5	73.3
octyl	0.97	0.97	1.9	33.7	34.7	34.7	17.7
nonyl	1.9	0.97	0.48	17.7	9.3	18.2	36.9
decyl	0.97	1.9	0.48	16	16.5	8.4	33.3
dodecyl	0.48	0.48	0.24	17.9	37.3	37.3	74.7
AMB	0.13	0.06	0.02	0.22	1.8	1.8	3.7
ITC	0.02	0.12	0.01	0.10	5.0	1.0	12.5

*MFC = MIC; Pb18, *P. brasiliensis* 18; Pb01, *P. lutzii* 01; Pb02, *P. brasiliensis* 02; MIC minimum inhibitory concentration); MFC, minimum fungicide concentration; MRC5, human normal pneumocyte cells; IC_*50*_, 50% inhibitory concentration; SI, selectivity index = (IC_*50*_/MIC); AMB, amphotericin B; ITC, itraconazole.*

### Cytotoxicity Assay with Protocatechuates

**FIGURE 2:**
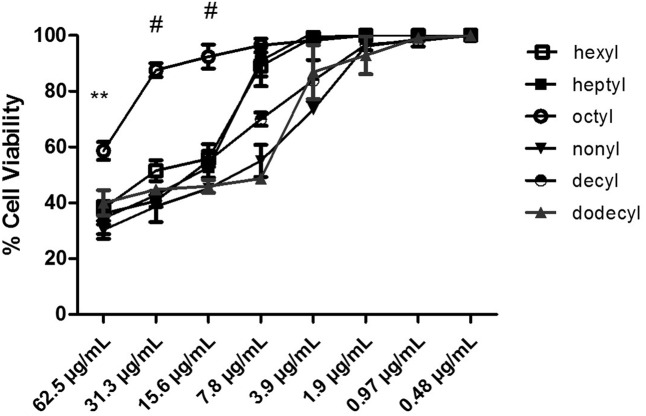
Human normal pneumocyte cells viability (%) in the presence of protocatechuates (hexyl, heptyl, octyl, nonyl, decyl, and dodecyl). The results are expressed as mean ± standard deviation (^#^*p* < 0.001, ^∗∗^*p* < 0.01).

A cytotoxicity assay was performed for all protocatechuates using the 0.4% sulforhodamine B method with the MRC5 cell line (human pneumocytes). The 50% inhibitory concentration (IC_50_) ranged from 0.48 to 7.8 μg/mL (**Table 1**). Dodecyl protocatechuate showed a better SI. The SI is the ratio of the concentration of a substance that induces 50% cell viability relative to the MIC of the same substance. For strain Pb02 in particular, a maximum SI (74.7) was observed (**Table 1**). The results are shown in **Figure 2**.

The MIC values demonstrate high cell viabilities for all the tested derivatives with some variation in cell viability: 99% for hexyl, 65% for heptyl, 98% for octyl, 96% for nonyl, and 98% for decyl and dodecyl. Thus, hexyl, nonyl, and dodecyl protocatechuate are the most promising substances because they are less toxic to the cell line, as shown in **Figure 2**. Statistical analyses indicated that the values were significantly different when comparing the compounds with the positive control (*p* < 0.001).

### Characterization of NLS

The protocatechuates, especially dodecyl, showed a very promising antifungal effect. However, the poor solubility of dodecyl led us to incorporate it into an NLS system in order to improve the pharmacological effect and cell viability and solubility.

**Table 2** details the diameter, index polydispersity, and the zeta potential values. The mean values for particle size, polydispersity, and the zeta potential of the nanoparticle were 151.22 nm, 0.16, and -58.30 mV for NLS and 147.82 nm, 0.13, and -68 mV.12 for NLS+dodecyl.

**Table 2:** Evaluation of particle size, polydispersity (PDI), and zeta potential of the NLSs.

Formulations	Diameter ± SD (nm)	PDI ± SD	Zeta potential ± SD
NLS	151.22 ± 0.60	0.16 ± 0.13	-58.30 ± 1.04mV
NLS+dodecyl	147.82 ± 0.43	0.13 ± 0.09	-68.12 ± 1.26mV

*The mean ± standard deviation (SD) is provided for 10 determinations of the formulations.*

**FIGURE 3:**
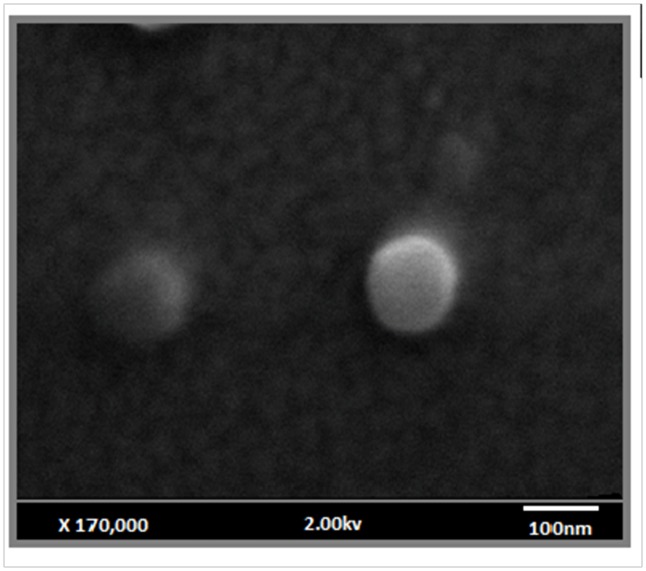
High resolution scanning microscopy images of the NLS loaded with dodecyl (NLS+dodecyl); 170,000× magnification.

**Figure 3** shows SEM images of NLS+dodecyl; as expected, isolated spherical shaped nanoparticles can be seen at 170,000× magnification.

### Minimum Inhibitory Concentration (MIC) of NLS+Dodecyl Protocatechuate

The MIC value of loaded dodecyl (NLS+dodecyl) was 0.03 μg/mL for both *P. lutzii* (Pb01) and *P. brasiliensis* 18 (Pb18) and was 0.06 μg/mL for *P. brasiliensis* 02 (Pb02). NLS+dodecyl exhibited a stronger antifungal activity against the three tested species, with increased MIC values (4- to 16-fold) compared to the MIC of free dodecyl. These results are presented in **Table 3**.

**Table 3:** Anti-*Paracoccidioides* activity and quantitative analysis of dodecyl and amphotericin B (AMB) incorporated into the NLS.

	Pb18 MIC	Pb01 MIC	Pb02 MIC
	(μg/mL)	(μg/mL)	(μg/mL)
Dodecyl	0.48	0.48	0.24
NLS+dodecyl	0.03	0.03	0.06
X increase	16	16	4
AMB	0.13	0.06	0.12

*MFC = MIC; Pb18, *P. brasiliensis* 18; Pb01, *P. lutzii* 01; Pb02, *P. brasiliensis* 02; MIC_*90*_, (90% minimum inhibitory concentration).*.

### Formation of the Adhesion Curve and Adhesion Inhibition Assay

**FIGURE 4:**
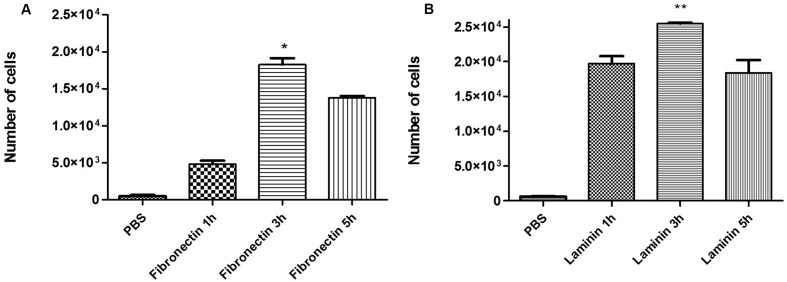
Adhesion curve to **(A)** the fibronectin matrix and **(B)** laminin matrix at 1, 3, and 5 h. The results are expressed as mean ± standard deviation; (^∗^*p* < 0.05, ^∗∗^*p* < 0.01) compared to all time points.

**FIGURE 5:**
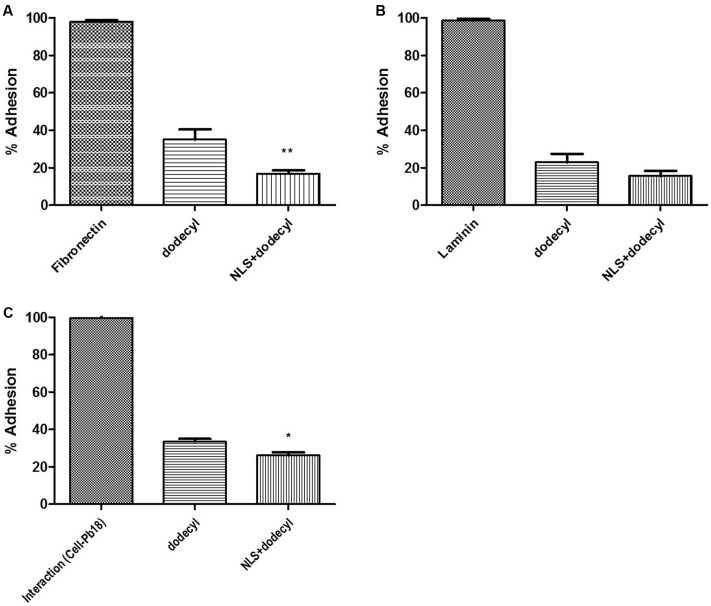
Inhibition of *P. brasiliensis* (Pb 18) adhesion to **(A)** the fibronectin matrix and **(B)** the laminin matrix by dodecyl and NLS+dodecyl. **(C)** Inhibition of *P. brasiliensis* (Pb 18) adhesion to cell line MRC5. The results are expressed as the mean ± standard deviation; (^∗^*p* < 0.05, ^∗∗^*p* < 0.01) compared to NLS+dodecyl and dodecyl.

The adhesion of *P. brasiliensis* (Pb 18) to ECM components (fibronectin and laminin) was assayed at 1, 3, and 5 h. The greatest adhesion of fungi to ECM was observed at 3 h, which was then used for subsequent experiments (**Figures 4A,B**). Inhibition of *P. brasiliensis* (Pb18) adhesion to ECM components (fibronectin and laminin) is shown in **Figures 5A,B**. Adhesion following 3 and 24 h of treatment with free dodecyl and dodecyl incorporated into the NLS system was used to establish the MIC values. Adhesion to the fibronectin matrix was 64 and 81% inhibited by dodecyl and NLS+dodecyl, respectively. The inhibition of adhesion to laminin was 75 and 82.4% for dodecyl and NLS+dodecyl, respectively. Inhibition of adhesion to the matrix proteins was especially strong when dodecyl was incorporated into the nanostructured system, which increased adhesion inhibition by 17%. The data showed significant differences when compared with the control without treatment (*p* < 0.05).

### Inhibition of *P. brasiliensis*–Cell Adhesion Interactions

**Figure 5C** demonstrates the inhibition of *P. brasiliensis* (Pb 18) adhesion to cell lines. Adhesion following 3 and 24 h of treatment with free dodecyl and dodecyl incorporated into the NLS system were used to establish the MIC value. Adhesion to cells was inhibited by 65 and 74% by dodecyl and NLS+dodecyl, respectively. Inhibition of cell adhesion was especially strong when dodecyl was incorporated into the nanostructured system, which increased adhesion inhibition by 9%. The data showed significant differences when compared with the control without treatment (*p* < 0.05).

### *In Vitro* Cytotoxicity Assay with NLS and NLS+Dodecyl

**FIGURE 6:**
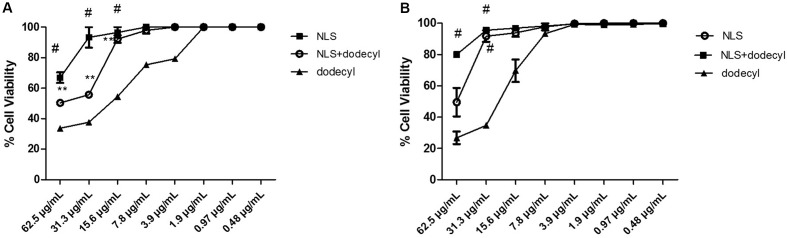
Percentage of Cell Viability results of the SNL (NLS), SNL+ dodecyl (loaded dodecyl) and free dodecyl, in **(A)** MRC5 and **(B)** HepG2. The results are expressed as mean ± standard deviation; (^#^*p* < 0.001, ^∗∗^*p* < 0.01), compared to dodecyl.

The potential cytotoxic effects of NLS and NLS+dodecyl were assessed with MRC5 (human pneumocytes) and HepG2 cells. NLS, NLS+dodecyl, and free dodecyl affected the cells in a concentration dependent manner. NLS decreased the toxicity of dodecyl in both line cells at higher concentrations (3.9 μg/mL for MRC5 and 15.6 μg/mL HepG2). The SI of NLS+dodecyl was 295 or 590, 4- to 16-fold higher compared to the SI free dodecyl (**Table 4** and **Figure 6**).

**Table 4:** Cytotoxicity and SI of dodecyl and amphotericin B (AMB) incorporated into the NLS.

	MRC5 IC_50_	SI (IC_50_/	SI (IC_50_/	SI (IC_50_/
	(μg/mL)	MIC Pb18)	MIC Pb01)	MIC Pb02)
Dodecyl	17.9	37.3	37.3	74.7
NLS+dodecyl	17.7	590.0	590.0	295
X increase	-	16	16	4
AMB	0.22	1.8	1.8	3.7

*MRC5 (human normal pneumocyte cells); SI (selectivity index) = (IC_*50*_/MIC), IC_*50*_ (inhibitory concentration 50%); MIC (minimum inhibitory concentration).*

### *In Vivo* Toxicity

**FIGURE 7:**
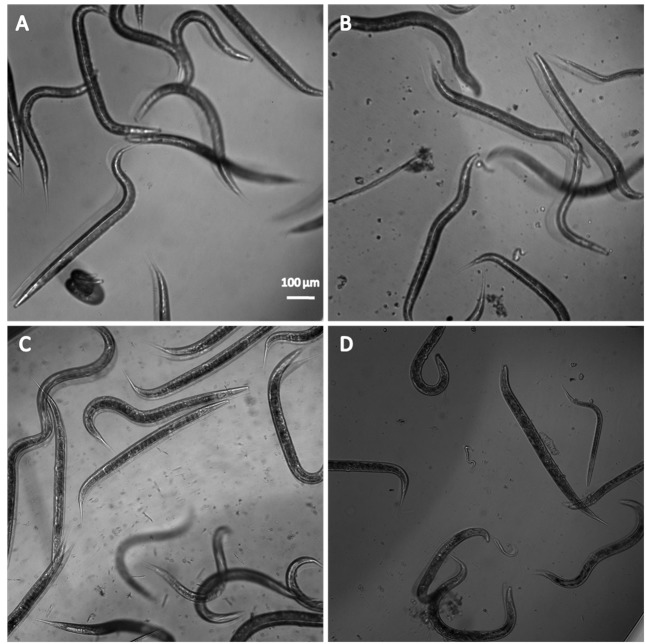
Representative image of *C. elegans* N2 **(A)** untreated, treated with **(B)** NLS, **(C)** dodecyl protocatechuate, and **(D)** and NLS+dodecyl.

To further elucidate the potential toxicity of free dodecyl protocatechuate or loaded dodecyl (NLS+dodecyl), we investigated the survival of *C. elegans* exposed to various compound concentrations (0.03, 0.48, 7.8, 15.6, 62.5, and 125 μg/mL). Exposure to dodecyl, NLS, and NLS+dodecyl did not affect worm survival at any concentration after 24 h; all worms exhibited a sinusoidal shape and mobility comparable to the untreated worms (**Table 5** and **Figure 7**).

**Table 5:** Survival percentage of *C. elegans* worms receiving no treatment (control) or treated with NLS (NLS), NLS+dodecyl, or dodecyl protocatechuate at different concentrations. Survival of treated worms is similar to that of the untreated controls.

Concentration (μg/mL)	Control	Dodecyl	NLS+dodecyl	NLS
	100			
0.03		100	100	100
0.48		100	100	100
7.80		100	100	100
15.60		100	100	100
62.50		100	100	100
125.00		100	100	100

## Discussion

Paracoccidioidomycosis treatment can include various antifungal agents such as AMB, azoles (including ITC), and sulfamidic acid; however, prolonged use of these drugs is toxic for patients. Studies with AMB have shown that this drug has several limitations including nephrotoxicity and cell death in patients (Richardson et al., 1988; Brajtburg et al., 1990; Hahn et al., 2003; Brime et al., 2004). Thus, there is a need for new therapeutic alternatives comprised of new nanocarrier substances that improve drug release and solubility and decrease compound toxicity (Kreilgaard, 2002; Patel et al., 2009).

In the current study, alkyl derivatives of protocatechuic acid were synthesized and tested for their antifungal activities against a number of *Paracoccidioides* spp. Our results show that hexyl, heptyl, octyl, nonyl, decyl, and dodecyl protocatechuate have strong antifungal effects against *P. brasiliensis* and *P. lutzii*; dodecyl protocatechuate showed a highest antifungal activity.

These findings indicate that dodecyl protocatechuate is a highly promising substance because it has a strong antifungal effect with minimal toxicity. Moreover, the results were correlated with the pharmacophore group (3,4-dihydroxybenzoic acid) and intermediate 12-carbon hydrophobic alkyl chain length probably allows increasing antifungal activity (Nihei et al., 2004).

Similarly, reported Nihei et al. (2003) evaluated a series of 3,4-dihydroxybenzoate alkyl protocatechuates and their fungicidal activity against *Saccharomyces cerevisiae* and concluded that the carbon chain plays a key role in antifungal and antioxidant activity.

Previous studies published by our group have shown similar findings for the activity of protocatechuic acid combined with fluconazole against *Trichophyton rubrum* and *T. mentagrophytes.* The evaluated compounds (pentyl, hexyl, heptyl, octyl, nonyl, and decyl) showed significant fungicidal effects with MICs ranging from 0.97 to 7.8 mg/L; high fungicidal activity was correlated with the hydrophobicity of the lateral chain of the compounds (Soares et al., 2014).

In addition, protocatechuic acid presented good cell viability in the cell lines tested; dodecyl presented the best cell viability and the highest SI, with values from 37.7 to 74.4. These data indicate that this compound is not toxic because it has an SI above the recommended SI (>10). Thus, dodecyl is more selective for the microorganism and possibly safer for the patient (Orme et al., 2001; de Freitas et al., 2014).

Based on these findings, dodecyl was incorporated into an NLS to increase its biological effects and facilitate its manipulation. The system can help to improve the pharmacokinetic and pharmacodynamic properties of compounds, including enhanced solubility, bioavailability, and stability, as well as protection against toxicity (Pannu et al., 2011; Cerpnjak et al., 2013). The system we developed has taken into account the toxicology of each formulation component with modification (Formariz et al., 2008, 2010).

The NLSs are formed from natural, semisynthetic, and synthetic lipids and contain many structures, such as cholesterol, phospholipids, and OS, which enable better delivery and entrapment because they are atoxic and biocompatible with the drug. However, in order to form a suitable matrix for the NLS, they must be adequately produced because they comprise the matrix of the drug and influence its behavior. The lipids chosen for incorporation must be potentially soluble, stable, and have low crystal formation (Müller et al., 2002; Hu et al., 2006; Pardeike et al., 2011).

Thus, we performed a physicochemical characterization of the NLS to determine the particle size (151.22 nm), PDI (0.16), and zeta potential (-58,30 mV). Dodecyl was subsequently incorporated into the system (NLS+dodecyl) resulting in a particle size of 147.82 nm, PDI of 0.13, and zeta potential of -68.12 mV. This particle size is within the 10–200 nm range, which are optimum values for the NLS and NLS+dodecyl (Formariz et al., 2005; Goyal et al., 2012). Light scattering analysis showed that the PDI values, calculated by dividing the mean droplet size by the mean number of droplets measured for NLS and NLS+dodecyl, were 0.16 and 0.13, respectively, indicating optimal good size distribution (<0.5) and reflecting system and NLS+dodecyl droplet size homogeneity (Janes and Alonso, 2003; da Silva et al., 2014). The zeta potential values for NLS and NLS+dodecyl were within the normal range (-30 to +30 mV). Negative charges are the result of considerable repulsion between surfaces, which is important to ensure nanodroplet stability because repulsive forces prevent the formation of nanoaggregates. Thus, the system and the drug nanocarrier that are incorporated should be standardized (Legrand et al., 1999; Schaffazick et al., 2003).

The SEM images of NLS+dodecyl revealed spherical shaped particles isolated, where the drug was inserted along the matrix homogeneously, observed in (**Figure 3**), respectively. Previous studies using a similar technique (ITC loaded into a NLS) observed spherical shape particles following pulmonary application (Pardeike et al., 2011).

The *in vitro* antifungal activity evaluation demonstrated higher antifungal activity for NLS+dodecyl and an MIC of 0.06–0.03 μg/mL for the *P. brasiliensis* and *P. lutzii* strains, constituting a 4- to 16-fold increase compared to the MIC range of free dodecyl (0.48–0.24 μg/mL). This increase was due to the efficiency of the delivery system and the capacity of the drug to be incorporated into the system matrix.

Subsequently, we evaluated the inhibitory effect of NLS+dodecyl on fungal adhesion to the host, which is the first step of infection. *Paracoccidioides* spp. can cause mycosis with a variety of clinical manifestations including localized and disseminated forms of the disease that can progress to lethality. Thus, the fungus must have developed mechanisms that enable it to adhere, invade, and pass through the barriers imposed by host tissues (Mendes-Giannini et al., 2000; Marcos et al., 2012). The ability of cells to interact with each other in an orderly manner depends on multiple interactions that allow for adhesion between cells and their extracellular environment; pathogen adherence depends on the recognition of carbohydrates and protein ligands on the surface of the host cell or ECM (Patti and Höök, 1994; Troyanovsky, 1999; Marcos et al., 2012).

In the present study, proteins present in the ECM, namely fibronectin and laminin, were used to examine the ability of free dodecyl protocatechuate and dodecyl incorporated into the nanostructured system to inhibit adhesion. Optimal *in vitro* adhesion of the fungus to fibronectin and laminin was observed at 3 h. Dodecyl and NLS+dodecyl exhibited 64% and 81% inhibition of adhesion to the fibronectin matrix, respectively, and 75 and 82.4% inhibition of adhesion to the laminin matrix, respectively. Dodecyl induces significant inhibition of adhesion and this inhibition increased up to 17% in the presence of the nanocarrier. To confirm these inhibition results we next tested the inhibition of *P. brasiliensis* (Pb18) adhesion to cells. Similar results were obtained, with 65 and 74% inhibition for dodecyl and NLS+dodecyl, respectively, a 9% increase with the NLS; these results indicate that the system potentiates the effect of dodecyl, inhibiting adhesion to the matrix and cells.

The MIC and inhibition of matrix and cell adhesion are significantly reduced for loaded dodecyl (NLS+dodecyl). This may be related to the fact that the system is formed by an oily phase, which contains lipids such as cholesterol and PC. These oily components act as permeation enhancers; contact surface density and droplet size are components that establish a greater area of contact with the surface of the mucosa providing the skin with a high concentration gradient and increasing the permeation of the system with the drug, thereby facilitating access of the drug to the microorganism (Peltola et al., 2003; Barot et al., 2012; Guimarães et al., 2014).

Therefore, the lipid composition of the NLS may have facilitated drug solubilization, thus significantly increasing antifungal activity. Many drugs exhibit increased antifungal activity upon incorporation with such systems; they become more easily soluble and consequently have increased pharmacological activity (Missner and Pohl, 2009; Bonifácio et al., 2015). A similar study of water-insoluble voriconazole incorporated into a microemulsion to improve solubility, resulted in increased permeation of the drug (favoring transdermal rather than dermal administration) against *Candida albicans* compared with the supersaturated solution of voriconazole (El-Hadidy et al., 2012).

In addition, our results can be attributed to the fact that the system can act as a drug reservoir enabling drug release from the internal phase to the outer phase during microorganism adhesion; this is facilitated by the oil phase of the system that is likely involved in the interaction with components of the fungal cell membrane, such as ergosterol, destabilizing its integrity and causing inhibition of adhesion and consequently parasite lysis and death (Cunha Júnior et al., 2003; Nirmala et al., 2013). A similar study showed that the NB002 nanoemulsion inhibited *C. albicans* and dermatophyte strains, suggesting that it could be acting via inhibition of ergosterol, leading to fungal death (Pannu et al., 2009).

The cytotoxicity results showed higher than 90% cellular viability for the system (NLS) and for NLS+dodecyl and did not show cytotoxic effects in cell lines (MRC5 and HepG2) even at high concentrations, in contrast to free dodecyl. These results demonstrate that NLS is a biocompatible formulation that is safe for use in eukaryotic cells; natural lipids are well tolerated by living organisms (Huang, 2008; Abbasalipourkabirreh et al., 2011; Oliveira et al., 2015). The SI is calculated by dividing the IC_50_ value by the MIC value. An SI ≥ 10 indicates that the compound can be applied at a concentration that is 10-fold higher than the MIC value without exhibiting cytotoxicity (Orme et al., 2001).

Our SI results revealed values >10. Loaded dodecyl (NLS+dodecyl), for example, presented an SI of 147, 16-fold higher than the SI of free dodecyl. Moreover, NLS+dodecyl has been shown to be more selective for the pathogen and is better tolerated by the host. This is because NLS+dodecyl contains cholesterol and other membrane lipids that promote the interaction with the cellular membrane, facilitating drug delivery to the place of action, without causing toxicity (de Freitas et al., 2014; Oliveira et al., 2015).

Subsequently, we tested free dodecyl protocatechuate and incorporated dodecyl (NLS+dodecyl) in a *C. elegans* model; neither compound exhibited a toxic effect on survival, indicating they were safe for use in the alternative *C. elegans* model. *C. elegans* was first used as an *in vivo* toxicity model for heavy metals (Williams and Dusenbery, 1988). More recently, toxicity assays using this nematode have been validated as good predictors for the adverse effects of many compounds, including antifungals and nanoparticles, in mammalian species (Okoli et al., 2009; Contreras et al., 2014; Rogers et al., 2015; Muhammed et al., 2016). This is important because of evidence suggesting that the basic physiological processes and stress responses in higher organisms (e.g., humans) and *C. elegans* are similar (Kaletta and Hengartner, 2006).

Based on our results we conclude that dodecyl exhibits antifungal activity, inhibits host-parasite adhesion in matrix and in cells without toxicity *in vitro* and *in vivo*, has normal SI values, and that incorporation into the NLS greatly improves these biological effects. Our results suggest that the NLS can be used as a strategy to solubilize drugs and improve antifungal activity against *P. brasiliensis* and *P. lutzii in vitro* and that NLS+dodecyl may constitute a very promising antifungal candidate for the treatment of PCM.

## Author Contributions

KM-A performed the tests: MIC, Cytotoxicity assay, Preparation of NLS, Characterization of the physicochemical properties of the nanoparticles, Adhesion of *P. brasiliensis* to artificial ECM and inhibition of adhesion in cell and writing of the manuscript. JS performed Toxicity assay in *C. elegans* model assay and writing of the manuscript. AV performed inhibition of adhesion in matrix and writing of the manuscript. JS performed the standardization of the adhesion and writing curve of the manuscript. MP prepared and characterized the Protocatechuate. MS prepared and characterized the Protocatechuate. CP preparated and characterized the Protocatechuate. LR professor guided in the Preparation and characterization of Protocatechuate and corrected the manuscript. VB professor oriented in the Preparation and characterization of Protocatechuate and corrected the manuscript. DdS professor oriented in the preparation and characterization of Protocatechuate and corrected the manuscript. MC professor of nanotechnology oriented in the preparation and characterization of the system and corrected the manuscript. MM-G professor guided in the biological tests and corrected the manuscript. AF-A professor guided in the biological tests and corrected the manuscript.

## Conflict of Interest Statement

The authors declare that the research was conducted in the absence of any commercial or financial relationships that could be construed as a potential conflict of interest.

## Abbreviations

AMB: amphotericin
ATCC: American type culture collection
CFU: Colony forming unit
CLSI M27-A3: clinical and laboratory standards institute M27-A3 Reference Method for Broth Dilution Antifungal Susceptibility Testing of Yeasts
DMSO: Dimethyl sulfoxide
ECM: extracellular matrix
EG/SEM: high resolution scanning microscopy
EtOAc: ethyl acetate
HepG2: human liver carcinoma cells
ITC: itraconazole
MIC: minimum inhibitory concentration
MRC5: human normal pneumocyte cells
NLS+dodecyl: dodecyl protocatechuate loaded-nanostructured lipid system
NLS: nanostructured lipid system
OS: sodium oleate
PBS: phosphate-buffered saline
PC: phosphatidylcholine
PCM: paracoccidioidomycosis
PDI: polydispersity index
SI: selectivity index.
